# The Effects of Storage Temperature on the Growth of *Vibrio parahaemolyticus* and Organoleptic Properties in Oysters

**DOI:** 10.3389/fpubh.2014.00045

**Published:** 2014-05-16

**Authors:** Meshack Fon Mudoh, Salina Parveen, Jurgen Schwarz, Tom Rippen, Anish Chaudhuri

**Affiliations:** ^1^Food Science and Technology Program, Department of Agriculture, Food and Resource Sciences, University of Maryland Eastern Shore, Princess Anne, MD, USA

**Keywords:** *Vibrio parahaemolyticus*, halophilic plate count, oysters, sensory, texture

## Abstract

During harvesting and storage, microbial pathogens and natural spoilage flora may grow, negatively affecting the composition and texture of oysters and posing a potential health threat to susceptible consumers. A solution to these problems would mitigate associated damaging effects on the seafood industry. The purpose of this study was to investigate the effects of storage temperature on growth of vibrios as well as other microbial, sensory, and textural characteristics of post-harvest shellstock Eastern oysters (*Crassostrea virginica*). Oysters harvested from the Chesapeake Bay, Maryland, during summer months (June, July, and August, 2010) were subjected to three storage temperatures (5, 10, and 20°C) over a 10-day period. At selected time intervals (0, 1, 3, 7, and 10 days), two separate samples of six oysters each were homogenated and analyzed for pH, halophilic plate counts (HPC), total vibrios, and *Vibrio parahaemolyticus* (*Vp*). Oyster meats shucked after storage were also organoleptically evaluated (acceptability, appearance, and odor). Texture analysis was performed using a texture analyzer on meats shucked from oysters held under the same conditions. The pH of the oyster homogenates showed no consistent pattern with storage time and temperature. The HPC (4.5–9.4 log CFU/g) were highest on day 7 at 20°C while olfactory acceptance reduced with time and increasing storage temperatures. The *Vp* counts increased over time from 3.5 to 7.5 log MPN/g by day 10. Loss of freshness as judged by appearance and odor was significant over time (*p* < 0.05). Toughness of oysters increased with storage time at 5 and 10°C from days 1 to 3 but was inconsistent after day 7. The results indicate that the length of storage and temperature had a significant effect on bacterial counts and olfactory acceptance of oysters but had an inconsistent effect on texture.

## Introduction

The Eastern oyster (*Crassostrea virginica*), commonly found in the intertidal zones of the eastern United States, has been traditionally harvested as a food source for thousands of years (1). Annual harvests of this species nationally were around 184.4 million pounds from 2005 to 2012 (2). Raw oysters are a natural source of minerals like calcium, iron, selenium, and zinc as well as high amounts of vitamin B12 and omega 3 fatty acids that improve neurological and cardiovascular health (3). Unfortunately, there is a high health risk for individuals who eat raw oysters because their natural flora may harbor human pathogens (4–6). Among the Gram-negative rods, *Vibrio parahaemolyticus* (*Vp*) is economically important because of its virulence. This halophilic bacterium (Family: *Vibrionaceae*) is commonly abundant in the estuarine waters worldwide and causes gastroenteritis in humans (7, 8). Human transmission of this pathogen is mainly through the consumption of raw, poorly cooked, or mishandled seafood including oysters. In addition to gastroenteritis with occasional bloody diarrhea, primary septicemia can occur in individuals with underlying chronic illness (8, 9). A number of *Vp* outbreaks associated with oyster consumption have been reported in the US and other countries in the past several years. Reports from the past decade indicate that *Vp* has accounted for approximately two-thirds of seafood borne infections in the US. These reports further indicated that *Vp* numbers in oysters and water increase with warmer temperatures (5, 9, 10). Several investigators (5, 6) confirmed that *Vp* growth was affected more by temperature than by salinity. The same authors also reported that oysters contained 233 times more *Vp* than the ambient water. Optimum growth of this pathogen occurs between 30 and 35°C with an upper limit of 45.3°C and in a range of 2–4% sodium chloride. Although *Vp* can grow over a broad pH range (4.8–11.0), the optimal range is pH 7.6–8.6 (8, 11).

In order to limit the growth of *Vp* in oysters, the National Shellfish Sanitation Program (12) established time–temperature regulations that restrict maximum exposure time of oysters to ambient temperatures. The regulation requires harvested oysters for raw consumption to be cooled to 10°C (50°F) or below within 10 h of harvest or less. A risk assessment conducted by the US Food and Drug Administration (13) found levels of *Vp* to be substantially influenced by post-harvest handling methods. The assessment pointed out that temperature abuse of oysters by improper refrigeration induces higher growth rates of *Vp*. This bacterium is also reported to multiply rapidly with a short generation time in both broth and seafood between 18 and 40°C (8, 11). Other research findings suggest that refrigerated storage should be considered a critical control point (CCP) in hazard analysis and critical control points (HACCP) plans for raw shellstock (4).

Temperature abuse may lead to pathogen growth as well as spoilage. It has been reported that the breakdown of glycogen, a high energy storage molecule in the oyster tissues, influences spoilage patterns of shucked oyster meats through fermentation. The resulting lactic acid accumulation and drop in pH favor the growth of Lactobacilli, Streptococci, and yeasts (14, 15). These changes may affect the sensory characteristics of oysters. Sensory evaluation of a food item sheds light on its consumer acceptability. Research indicates that when homogenated oyster meat and liquor pH drop into the range of 5.6–6.1 sensory scores decline from good to stale, or stale to sour or putrid for pH between 4.9 and 5.3, and then to an “advanced decomposition” stage for pH ≤ 5.0. This relationship of shucked oyster meat and liquor has been established as a basis for evaluating its wholesomeness (14). Lorca et al. (4) compared oyster bacterial counts with sensory perception. Olfactory acceptability and storage time were negatively correlated, and the former was below 40% when *Vibrio vulnificus* (*Vv*) growth was at its highest. These researchers only measured the olfactory acceptability of shellstock oysters.

Complimenting sensory information with instrumental analyses can improve the accuracy of the observations (16). Texture is an important characteristic of food quality especially in aquatic foods (17) and can provide valuable information about food storage and handling conditions. There is inadequate science-based information about the effects of storage temperature on the growth of *Vp*, and corresponding physical and sensory properties of shellstock (live) oysters that is relevant to the shellfish industry and consumers. The objective of this study was to address these data gaps.

## Materials and Methods

### Oyster samples

Oyster samples (*C. virginica*) were collected from the Chesapeake Bay, MD, USA, during the summer months (June–August 2010). The oyster samples were then placed in pre-labeled clean plastic bags, transferred to an insulated chest, and bubble wrapped. Ice bags were placed on the wrap to prevent direct contact of samples with ice. The temperature was monitored during shipping to verify that it was <10°C, which is the minimum temperature for growth of *Vp* (11). The samples were transported to the laboratory for subsequent analyses. In addition, harvest water temperature and salinity were measured in the upper 0.5 m of the surface water using a dissolved oxygen-conductivity meter (Model 85, Yellow Springs Instrument Co., Yellow Springs, OH, USA). All microbiological assays were initiated within 4–6 h of sample collection (5). For this study, sets of 50 oysters were stored at 5, 10, and 20°C to mimic typical conditions of oyster handling, where 5°C is the desired refrigeration temperature, 10°C the abusive refrigeration temperature, and 20°C reflecting ambient temperatures often occurring during oyster harvest. At selected time intervals (0, 1, 3, 7, and 10 days), two separate subsets of six oysters each were analyzed for halophilic plate counts (HPC), total vibrios, and *Vp* counts. All analyses were performed on viable oysters. The samples for microbiological analyses were removed, and the pH of the remaining oyster homogenates was determined for each sample using a calibrated pHTESTR™(Oakton Instruments, IL, USA).

All experiments were carried out in replicates of three, and for each replication, sample analyses were performed in duplicates.

### Microbial analyses

At each selected time interval, two sub-samples (six oysters/sample) were scrubbed, rinsed, shucked, and then diluted 1:1 (w/w) with sterile phosphate-buffered saline [PBS, 76.5 g NaCl, 0.724 g of anhydrous Na_2_HPO_4_, 0.21 g of KH_2_PO4/l, pH 7.4 (Sigma^®^, St. Louis, MO, USA)]. The oyster homogenates were blended on high speed for 90 s in a sterile Warring^®^ blender. A 1:10 dilution (w/w) of oyster homogenate was obtained by mixing 20 g of homogenate and 80 g of PBS. Serial dilutions were then prepared in PBS (v/v basis). One hundred microliters and 1 ml samples from each dilution were inoculated into trypticase soy agar (TSA) + 2.5% NaCl plates for HPC (Difco Laboratories^®^, Detroit, MI, USA) and 10-ml aliquots of alkaline peptone water (1× APW, at pH 8.5) three-tube most probable number (MPN) series, respectively. TSA plates were read for HPC after 48 h of incubation at 35°C. For MPN, after 24 h of incubation, one loop of inoculate from the top 1 cm of fluid of each APW tube (5) was streaked onto thiosulfate citrate bile salts (TCBS) agar (Difco^®^) plates and incubated for 24 h at 35°C. TCBS plates were checked for any growth to indicate total *Vibrio* counts. The growth data were combined and total *Vibrio* MPN/g was calculated using a three-tube MPN table (4, 18). After 24 h incubation, all turbid APW tubes were analyzed by q-PCR for the detection of *Vp*. From each turbid tube, 200 μl of each oyster enrichment broth was boiled for 10 min and then plunged into ice. The cooled aliquots were spun at 10,000 × *g* for 2 min to pellet cellular debris and a 2-μl portion of the supernatant was used as template in the q-PCR assay to detect the presence of the *tlh* gene. All primer and probe sequences, reaction conditions, cycling, and analysis parameters were used as previously described (19).

### Sensory evaluation

The sensory evaluation (appearance and odor) was performed with the help of 25 semi-trained panelists from the University of Maryland Eastern Shore campus, Princess Anne, MD, USA (males-11; females-14; age group: 20–60) using 15 cm semi-structured category scale for freshness. All panelists were trained to mark horizontally across the scale lines to indicate their judgment of the oyster. The left hash mark on each category scale indicated “*very fresh*” while the right hash mark indicated “*not fresh*.” During the evaluation, every panelist was provided with three shucked oysters with its liquor representing the three different storage temperatures at each selected time interval. Oyster samples were coded with three-digit random numbers and presented to panelists in random balanced order under fluorescent light at room temperature (4). The panelists were asked to evaluate the samples for degree of freshness by appearance and odor on the 15-cm scale. Similar scales are used to quantify specific attributes in a food item (20). Panelists were also asked to indicate yes or no if they would eat each oyster presented.

### Texture analyses

Texture was analyzed by measuring the resistance to shear using a TA-XT2 texture analyzer (Stable Micro System, Godalming, England) with a 5-kN load cell. The shucked oyster meat was placed on the instrument platform under a knife blade (3 mm thick with a 45° chisel end), compressed, and sheared to measure the force of penetration (resistance to shear). Great care was taken to position the oyster samples so that the blade contacted the meat at its thickest point. The crosshead speed was set at 1 mm/s, the distance set for the blade to stop within 1 mm of the platform after which it returned automatically to the start position, and the test was triggered by a 5-N contact force.

The force–time graphs were recorded and analyzed using the Texture Expert for Windows (version 5.0.9.0, Stable Micro Systems, Ltd., UK.). The peak force was expressed as maximum resistance to shear (N) and the area under the curve as total work (N × mm) and is a measure of toughness of the oysters (17).

### Statistical analyses

Before analyses, geometric means of all data were normalized using log or square root transformation methods (as appropriate) to reduce numerical variability in the dataset. A two-way between groups, analysis of variance (ANOVA) was used to evaluate the role of storage temperatures and time (days) on microbiological counts in oyster samples (*p* < 0.05). Pearson correlation was used to evaluate the data from sensory experiments. Panelist marks on the scales were translated as percent of full scale (0–100, with 100 indicating the highest level of freshness) and analyzed using one-way ANOVA, Tukey HSD all-pair-wise comparisons test and Pearson correlation (*p* < 0.05). All statistical analyses were done using Statistica^®^ (StatSoft, Inc., OK, USA. Statistica data analysis software system, version 9.0. www.statsoft.com).

## Results

### Oyster quality and pH

The pH values of oyster meat and liquor stored at 5, 10, and 20°C for 10 days are shown in Table 1. The pH appeared to drop from days 0 through 3 for oysters stored at 5 and 10°C but then increased. These apparent changes in pH were not statistically significant (*p* > 0.05).

**Table 1:** **Changes in pH values of oysters during a 10-day storage period**.

Storage time (*d*)	Storage temperature (°C)	Average pH ± SD
0	–	6.55 ± 0.19
1	5	6.42 ± 0.02
	10	6.44 ± 0.01
	20	6.43 ± 0.03
3	5	6.43 ± 0.05
	10	6.42 ± 0.01
	20	6.46 ± 0.08
7	5	6.46 ± 0.03
	10	6.45 ± 0.02
	20	6.36 ± 0.16
10	5	6.57 ± 0.03
	10	6.62 ± 0.15
	20	6.08^a^

*^a^ There was only one value for this set of data as the other oysters perished*.

### Halophilic plate counts

**Figure 1:**
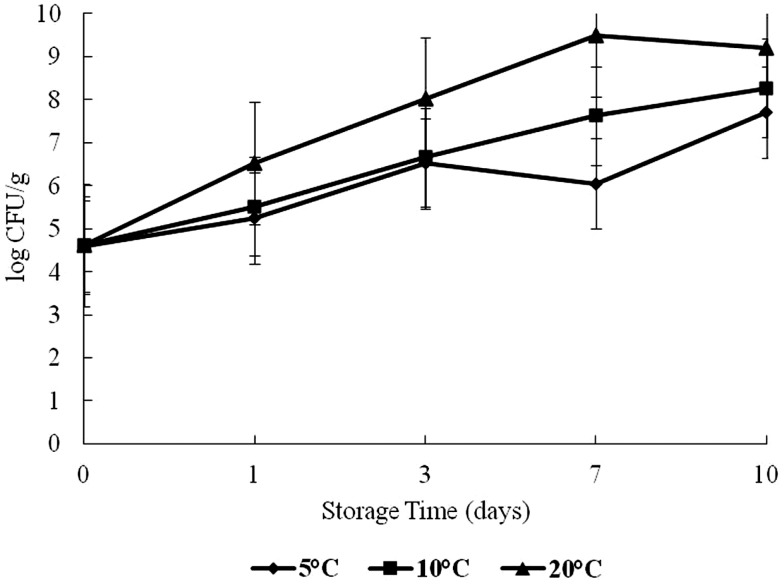
**Halophilic plate count (HPC) of oysters during a 10-day storage period at 5, 10, and 20°C**.

Halophilic plate counts of oysters were highest on day 7 at 20°C and on day 10 at 5 and 10°C during storage, with a log range of about 4.5–9.4 log CFU/g (Figure 1). The HPC increased significantly as time and temperature increased (*p* < 0.05).

### Total vibrios

**Figure 2:**
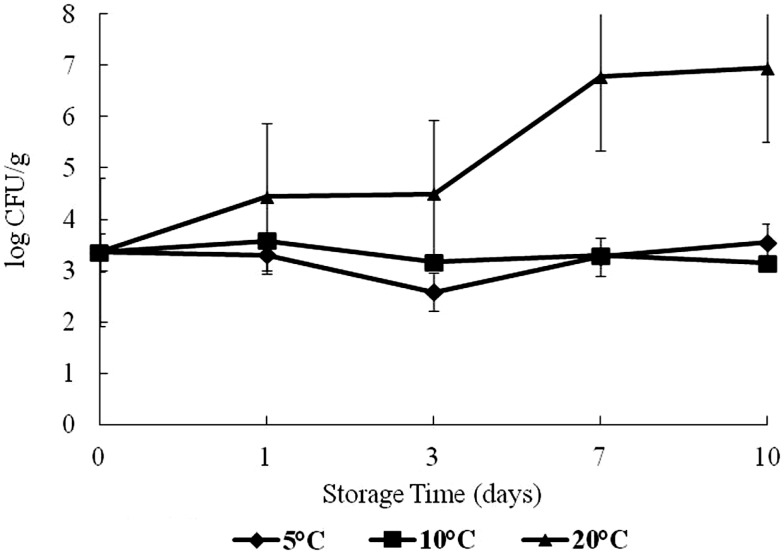
**Levels of total vibrios during 10 days of storage at 5, 10, and 20°C**.

Total vibrio*s* increased over time at 20°C (Figure 2). The highest total *Vibrio* count was observed on day 10 at 20°C storage, with about 3.4–7.5 log CFU/g difference from day 0. There was no statistical difference (*p* > 0.05) in the growth of total Vibrios at 5 and 10°C in this study. The effect of time and temperature on *Vibrio* counts was statistically significant at 20°C (*p* < 0.05).

### *Vibrio* *parahaemolyticus*

**Figure 3:**
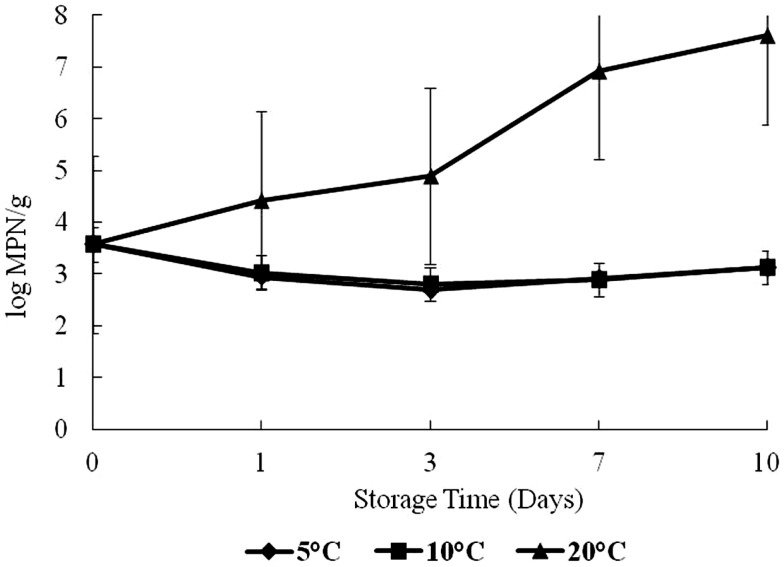
**Levels of *Vibrio parahaemolyticus* during 10 days of storage at 5, 10, and 20°C**.

On day 0, the level of *Vp* was 3.5 log CFU/g in oyster samples (Figure 3). Growth trends for *Vp* were similar as for total vibrios. *Vp* levels increased significantly (*p* < 0.05) over time with storage temperatures (Figure 3). This study observed a dramatic increase after day 3 at 20°C. The highest level of *Vp* counts was observed on day 10 at 20°C storage, with about a 3-log increase from day 0. No increase was observed in *Vp* counts at 5 and 10°C.

### Acceptability to consume oysters

**Figure 4:**
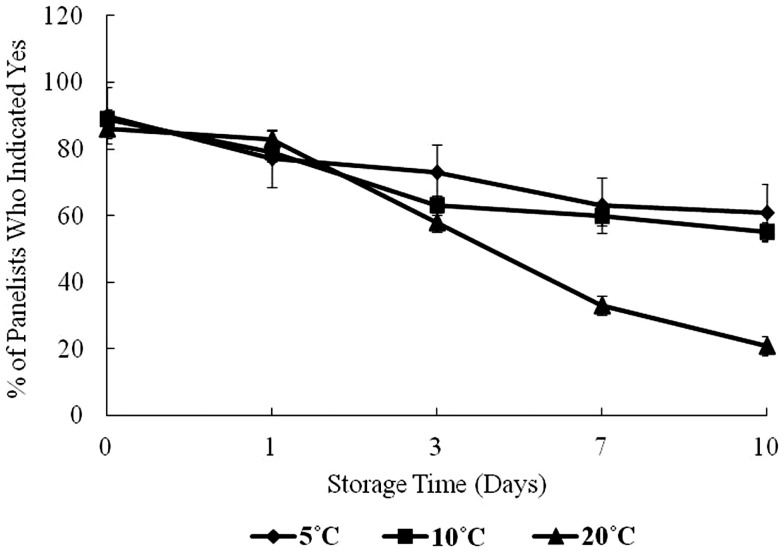
**Sensory evaluation of oysters under controlled conditions over a 10-day storage period at 5, 10, and 20°C, percent of panelists indicating yes, they would consume the oyster**.

Figure 4 shows the result of panelists’ organoleptic response to samples during the 10-day storage period. At day 0, approximately 90% of panelists indicated “yes” to their willingness to consume the oysters. From days 1 to 10, the percentage of panelists willing to consume the oysters dropped steadily.

At 5 and 10°C storage temperatures, the acceptance dropped to about 60% of panelists who were willing to consume the oysters on day 10. At 20°C, sensory acceptance dropped dramatically throughout the study to about 30%. In this study, all microbiological variables showed a positive correlation with storage time, and as storage time and temperature increased, the olfactory acceptability of oysters decreased.

### Response of panelists to visual appearance and odor of oysters

**Figure 5:**
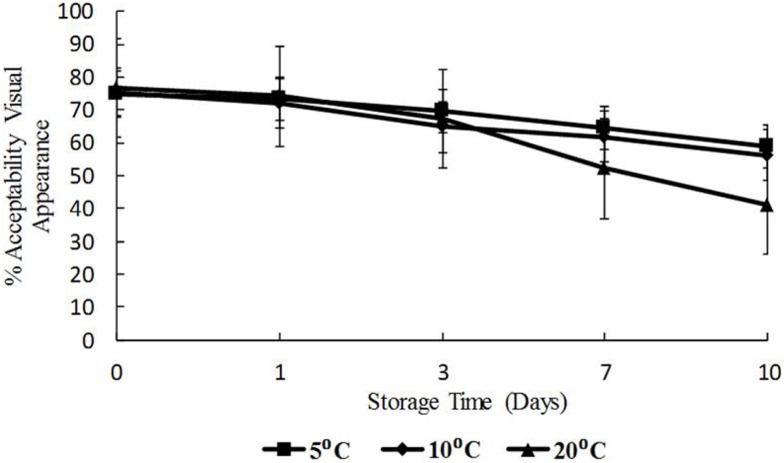
**Freshness as determined by visual appearance with time (days) and temperature (5, 10, and 20°C), shown as percent of full scale, 100 = fresh, 0 = not fresh**.

**Figure 6:**
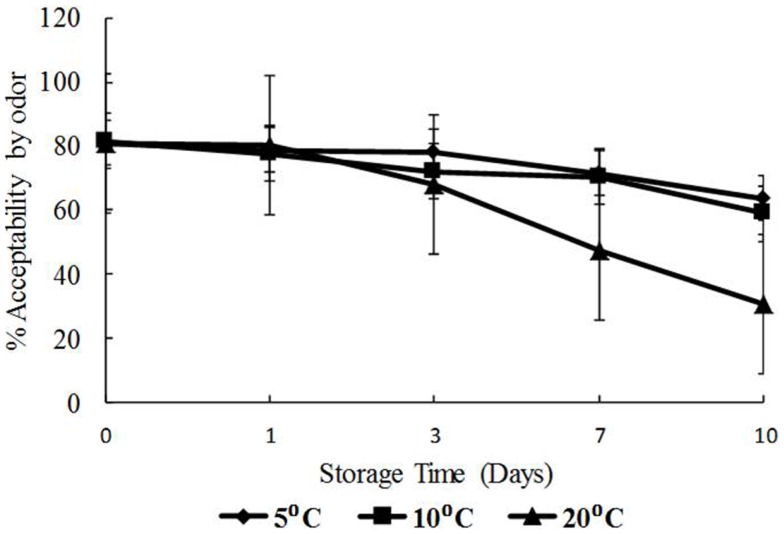
**Freshness as determined by odor with time (days) and temperature (5, 10, and 20°C), shown as percent of full scale, 100 = fresh, 0 = not fresh**.

The data for visual appearance and odor were strongly correlated (*p* < 0.05) indicating that as the temperature increased, both appearance and odor deteriorated. Odor and appearance freshness scores declined significantly from days 7 to 10 and at 10 and 20°C (*p* < 0.05) (Figures 5 and 6). A separate pair-wise comparison test for odor and time with temperature increase, showed a significant decrease in fresh odor only from days 7 to 10 (*p* < 0.05). In this study, HPC significantly increased with temperature and time, and the decline in freshness judged by odor and appearance followed the same trend.

### Textural analyses

**Figure 7:**
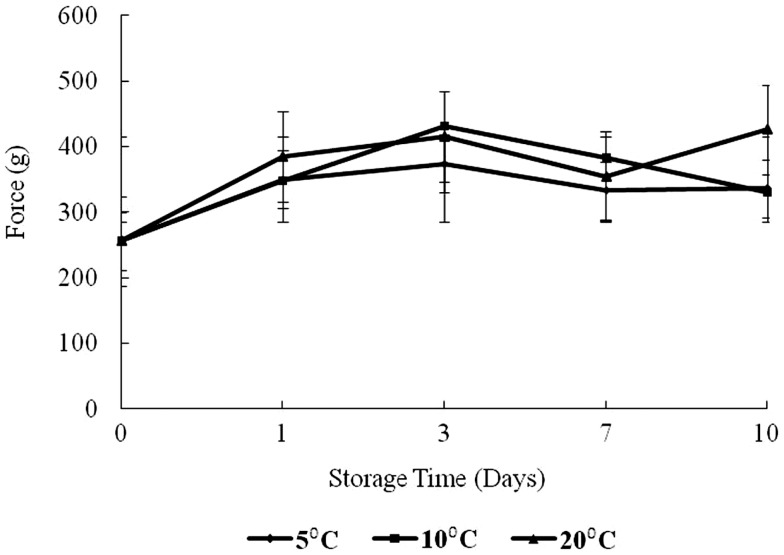
**Changes in force versus storage time (days) and temperature (5, 10, and 20°C)**.

**Figure 8:**
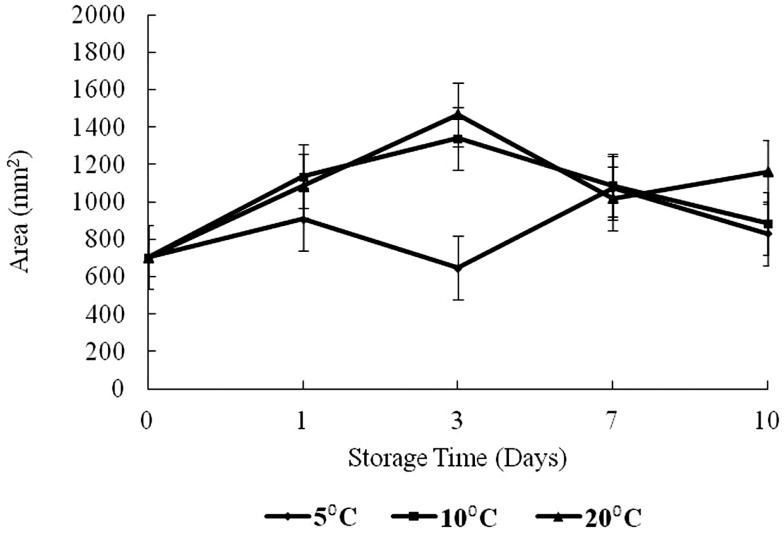
**Changes in area versus storage time (days) and temperature (5, 10, and 20°C)**.

The results of texture analysis of oysters stored for 10 days under the various temperature conditions (5, 10, and 20°C) are shown in Figure 7. The peak force increased for all three storage temperatures from days 0 to 3 was not significant. In contrast, the increases in peak force up to day 3 for the 10 and 20°C treatments and the increase for the 5°C treatment between days 0 and 1 were significant (*p* < 0.05). The 10°C treatment showed a steady decrease in peak force from days 3 to 10, while the 20°C treatment decreased to day 7. The apparent peak force increase on day 10 at 20°C was not significant. At 5°C the peak force decreased to day 7, after which an apparent slight but insignificant increase was observed (*p* > 0.05). The total work defined by the area under the shear resistance curve is shown in Figure 8. The increase of peak force was observed at day 3 with a subsequent decrease at day 7.

## Discussion

The present study is the most comprehensive and first report to date on the effects of storage temperature on the growth of *Vp*, and physical and sensory properties of live oysters. Oysters were harvested from the Chesapeake Bay during the warmer months and stored over a range of temperatures to be representative of industry and consumer practices.

It has been reported that as the pH of stored shucked oyster meats drop to five or below, the meat is considered spoiled and unfit for consumption (14). The results on pH variations in this study are consistent with the works of Lorca et al. (4) as well as Hunter and Linden (14). Lorca et al. (4) observed that the pH of the shellstock samples never fell below an average of 5.9, and in this study, the lowest pH was 6.08. These were live marine organisms held out of water and their physiology likely reflected their starved condition. Glycogen would be utilized quickly and not readily restored. Unlike shucked oyster meats, the absence of a continued downward trend in pH during the shellstock oyster storage might be attributed to the near lack of fermentative spoilage in shellstock. From this study, pH does not appear to be a useful indicator of bacterial growth in live oysters considering the observed growth of *Vp*.

The combined effect of time and storage temperature on HPC was statistically significant (*p* < 0.05). Despite the general increase in HPC, there was an observed drop from days 3 to 7 for the 5°C treatment which was statistically insignificant (*p* > 0.05). The 20°C treatment also showed a decrease from days 7 to 10. Bacteria competing for nutrients may have caused this observed drop. Perhaps, those cells that survived the competition regained vigor to grow. The growth pattern of HPC in this study finds support from studies conducted by Hood et al. (21) and Lorca et al. (4). These authors observed a similar effect in both shellstock and shucked oysters stored at selected temperatures (2, 8, 20, and 30°C).

Total *Vibrio* counts remained at approximately 3.3 log CFU/g or below at 5 and 10°C indicating that low temperatures can suppress total *Vibrio* growth. This study corroborates the findings of Cook and Ruple (15) as well as Lorca et al. (4). They had reported similar total *Vibrio* results with post-harvest shellstock oysters stored at 7, 13, 21, 22, and 30°C for a 10-day period in oysters.

In this study, average harvest water temperature and salinity were 22.8 ± 2.71°C and 21.47 ± 5.11 ppt, respectively. These parameters are consistent with the results of a previous study in the Chesapeake Bay (5). These researchers attributed the highest levels of *Vp* to warm water temperatures and relatively low salinities. The detection of *Vp* agrees with research findings that have reported higher *Vp* levels in oysters during the warmer months (5, 6).

*Vibrio parahaemolyticus* levels over time increased with storage temperatures. Lorca et al. (4) reported similar results for *Vv* in shellstock oysters stored at 7, 13, and 21°C for a 10-day period. Cook and Ruple (15) reported a similar rise in *Vibrio* levels in post-harvest shellstock oysters stored at 22–30°C. Recently, Parveen et al. (11) reported that *Vp* multiplied rapidly when oysters were stored at 15°C or above, with no growth at 5 and 10°C. The results of this study suggest that storage of oysters at or below 10°C is effective for preventing *Vp* growth in shellstock oysters.

In this study, the “freshness score” by panelists gradually decreased during storage at different temperatures. Relating the bacterial growth to the response of the panelists showed a high level of agreement. Willingness to consume oysters was inversely related to the rise in HPC and *Vp* over time, especially at the highest temperature, 20°C. These results are consistent with the results of Cao et al. (22) in a study of Pacific oysters (*Crassostrea gigas*) at different storage temperatures. It was also observed that appearance and odor of oysters were negatively correlated to bacterial counts, storage time, and elevated temperatures. However, these results also show that simple olfactory analysis of raw shellstock may not be adequate to prevent oyster-associated *Vp* illness. The authors suggest that extended high temperature storage (20°C) could pose a health threat to a minority of consumers willing to consume them (30% of panelists willing to consume on day 7).

The pH of the oysters in this study never fell below 6.08, indicating little loss of quality resulting from the growth of fermentative bacteria. As previously discussed, HPC growth was higher than *Vp* by a minimum of 2 logs at all storage conditions. It was also noted that at 20°C, the levels of *Vp* were significantly higher than at lower temperatures (with an increased growth of about 3.8 MPN/g). Storage of shellstock at elevated temperatures and time stimulated *Vp* growth without altering the chemical quality of the oysters (as indicated by pH). It should be understood that as both pH and olfactory acceptability of oysters stored at elevated temperatures fell within the accepted range, there was a possibility of concurrent elevated levels of *Vp* that could present a potential undetected health threat to consumers.

Peak force is a measure of hardness of a product tested while the area or total work represents toughness (17). Dobraszcyk and Vincent (23) define the area as the energy required to fully compress the tissue up to the point of failure and reflects the toughness or extensibility of the tissue. Aussanasuwannakul et al. (17) did not observe a change in peak force of refrigerated salmon raw filets (for 3 and 7 days). Subsequent freezing of the filets for 30 days resulted in an apparent reduction in peak force, though insignificant. The authors also observed a slight increase in total work from 3 to 7 days of refrigerated storage, but this change was also insignificant. By contrast in broiler breasts, Lee et al. (24) determined that tenderness (measured by Meullenet–Owens razor) shear force and energy decreased during long-term frozen storage (4 months) due to muscle shrinkage by dehydration. The increase of peak force observed at day 3 in the present study might have been caused by an initial loss of moisture. The decrease at day 7 could have been caused by catabolic processes or proteolytic degradation of myofibrillar structures and connective tissue networks which reduce shear resistance as discussed by Ashie and Simpson (25). It is noteworthy to mention that at the time of writing this manuscript, the authors could not find any comparable textural study on oysters in existing databases.

It has to be noted that the oysters used in this study were alive, although they were not stored in water. In living tissue, there is a constant state of equilibrium between the plasma protein, the amino acids of the blood, and the tissue proteins (26). In this study, it seems that constituent equilibrium could not be maintained in the live oysters during storage, producing the observed changes in peak force. However, further studies on the chemistry of proteins, native enzymes, and moisture migration at different storage temperatures over time would improve understanding of texture changes in shellstock oysters.

The area under the force versus time curve represents the energy required to fully compress the tissue up to the point of failure and reflects the toughness or extensibility of the tissue (23). In this study, the area was observed to increase to day 3 for 10 and 20°C treatments (*p* < 0.05). The area for 10°C oysters continued with a decrease to day 10 while for the 20°C treatment, there was also an observed decrease from days 3 to 7 followed by an increase in area on day 10. For 5°C, the total work measured did not show any obvious trend, increasing to day 1, and then decreasing to day 3 followed by an increase to day 7 with virtually no change to day 10. The initial increase seen at all temperatures is likely the result of dehydration. The differences in total resistance from days 3 to 10 could reflect the enzymatic degradation of proteins. These oysters were live animals and physiologically active, except possibly near the end of the storage period at 20°C.

In conclusion, all microbiological variables showed a positive correlation to storage time and temperature, and as storage time and temperature increased, the olfactory acceptability of oysters decreased. However, the pH of oyster homogenate did not show any significant decrease in this study. The authors suggest oyster storage temperatures at or below 10°C will be effective in controlling *Vp* growth, and it should be considered a CCP in HACCP plans used by the oyster industry. In addition, texture indicators showed an increase and then decrease in toughness of the oysters as storage temperature and time increased.

The result of this study should assist risk managers and seafood industry management in developing guidelines for oyster storage prior to their consumption. These findings should assist regulatory officials and the food industry in their review of policies on the handling of oysters. Also, the results of this research could assist risk assessors and the seafood industry in designing interventions to control the risk of vibrios to human health.

The authors suggest further research on glycogen levels and other physiological factors that may shed light on the role of microbial growth parameters in stored shellstock oysters. Also beyond the scope of this study was the enzymatic and protein chemistry of shellstock oysters under different storage conditions.

## Conflict of Interest Statement

The authors declare that the research was conducted in the absence of any commercial or financial relationships that could be construed as a potential conflict of interest.
